# A Foraging Mandala for Aquatic Microorganisms

**DOI:** 10.1038/s41396-018-0309-4

**Published:** 2018-11-16

**Authors:** Vicente I. Fernandez, Yutaka Yawata, Roman Stocker

**Affiliations:** 1 Department of Civil, Environmental and Geomatic Engineering, Institute of Environmental Engineering, ETH Zurich, Zurich, Switzerland; 2 Faculty of Life and Environmental Sciences, University of Tsukuba, Tsukuba, Japan

**Keywords:** Microbial ecology, Microbial ecology, Water microbiology

## Abstract

Aquatic environments harbor a great diversity of microorganisms, which interact with the same patchy, particulate, or diffuse resources by means of a broad array of physiological and behavioral adaptations, resulting in substantially different life histories and ecological success. To date, efforts to uncover and understand this diversity have not been matched by equivalent efforts to identify unifying frameworks that can provide a degree of generality and thus serve as a stepping stone to scale up microscale dynamics to predict their ecosystem-level consequences. In particular, evaluating the ecological consequences of different resource landscapes and of different microbial adaptations has remained a major challenge in aquatic microbial ecology. Here, inspired by Ramon Margalef’s mandala for phytoplankton, we propose a foraging mandala for microorganisms in aquatic environments, which accounts for both the local environment and individual adaptations. This biophysical framework distills resource acquisition into two fundamental parameters: the search time for a new resource and the growth return obtained from encounter with a resource. We illustrate the foraging mandala by considering a broad range of microbial adaptations and environmental characteristics. The broad applicability of the foraging mandala suggests that it could be a useful framework to compare disparate microbial strategies in aquatic environments and to reduce the vast complexity of microbe-environment interactions into a minimal number of fundamental parameters.

## Introduction

Aquatic environments harbor one of the Earth’s largest pools of organic carbon [1] and aquatic microorganisms are the tireless scavengers who mediate the circling back of dissolved organic carbon into the higher food chain and the atmosphere – a material flow called the microbial loop [2]. This global carbon flow, with a flux estimated at 50 Gt/year [3], occurs through microorganisms encountering and engaging organics – whether molecules, patches, or particles – at the microscale. The microbial hunt for resources – or ‘foraging’ – is directly shaped by the small length scales of both the organisms and the resources. Physical processes, particularly mass transport, Brownian dynamics, and low Reynolds number fluid flow, govern the microscale environment and exert constraints on microbial foraging adaptations.

The water column of aquatic environments supports microorganisms with multitudes of uptake mechanisms, sizes, morphologies, and behaviors. Some microorganisms sink while others are buoyant, some are motile while others cannot swim, some adhere to surfaces while others remain planktonic, some form biofilms on surfaces, some exude enzymes to break down substrates, and many metabolize a broad diversity of compounds among the rich chemical palette of resources available in the water column. Studies of aquatic microorganisms have frequently focused on understanding the nature of these adaptations and their impact on the growth and survival of the microorganisms that exhibit them [4]. This extensive body of work has resulted in the discovery of unique physiological and behavioral adaptations of aquatic microorganisms and has revealed the diversity of their nutrient acquisition strategies. However, still missing is an overarching framework that guides our understanding of the effectiveness of diverse foraging adaptations and how these adaptations relate to the properties of the resource landscape and the physical environment. Such a framework would underpin the ability to compare the performance of different foraging adaptations and to better understand competition for resources among microorganisms in an often resource-poor water column. The purpose of this perspective is, then, to outline a candidate biophysical framework that condenses the large amount of complexity uncovered by previous research into two fundamental variables.

The impact of microbial adaptations on the growth of organisms cannot be disentangled from the environment in which organisms acquire resources. To capture this coupling, we propose a new biophysical framework that, inspired by Ramon Margalef’s work on phytoplankton [5, 6], we refer to as a foraging mandala. The term mandala refers to a graphical representation of the order in a given world, and we use the term to simultaneously encompass the diversity and the hidden order found among environmental and biological factors that shape microbial foraging in the aquatic environment. The foraging mandala maps aquatic microorganisms onto a space consisting of two fundamental parameters of resource foraging: the time a microorganism takes to find a new region of elevated resources (‘search time’) and the yield that a microorganism obtains from the encounter with one such resource patch (‘growth return’). As will be seen, we interpret ‘search time’ and ‘patch’ very broadly, encompassing cases that would not traditionally be considered foraging, including bacteriophage-host interactions, horizontal transmission of endosymbionts, and oligotrophic bacteria in well mixed environments. For instance, non-motile oligotrophs can “search” the water column purely by passive diffusion to encounter – and exploit for their growth – minute “patches” that can be as small as single molecules of organic carbon, even when these are scarcely and homogenously distributed in the water column.

The foraging mandala encompasses all aquatic microbes that interact with dissolved or solid chemical resources, and other behaviors that can be cast as foraging. Some resource interactions, however, such as that of photoautotrophs with sunlight, are not easily incorporated into a foraging framework. While we will often draw examples from heterotrophic bacteria, the biophysical processes we cover extend to a multitude of aquatic microorganisms, including phytoplankton, autotrophic bacteria, and viruses. In this framework, adaptations are accounted for solely in terms of their impact on the search time and the growth return axes of the foraging mandala. We propose that this approach provides an immediate, comparative overview of the role and effect of different nutrient acquisition strategies among aquatic microorganisms and aids in evaluating the competition among microorganisms that use different strategies, the conditions favouring diversity, and the impact of environmental changes.

## The foraging mandala

**Fig. 1:**
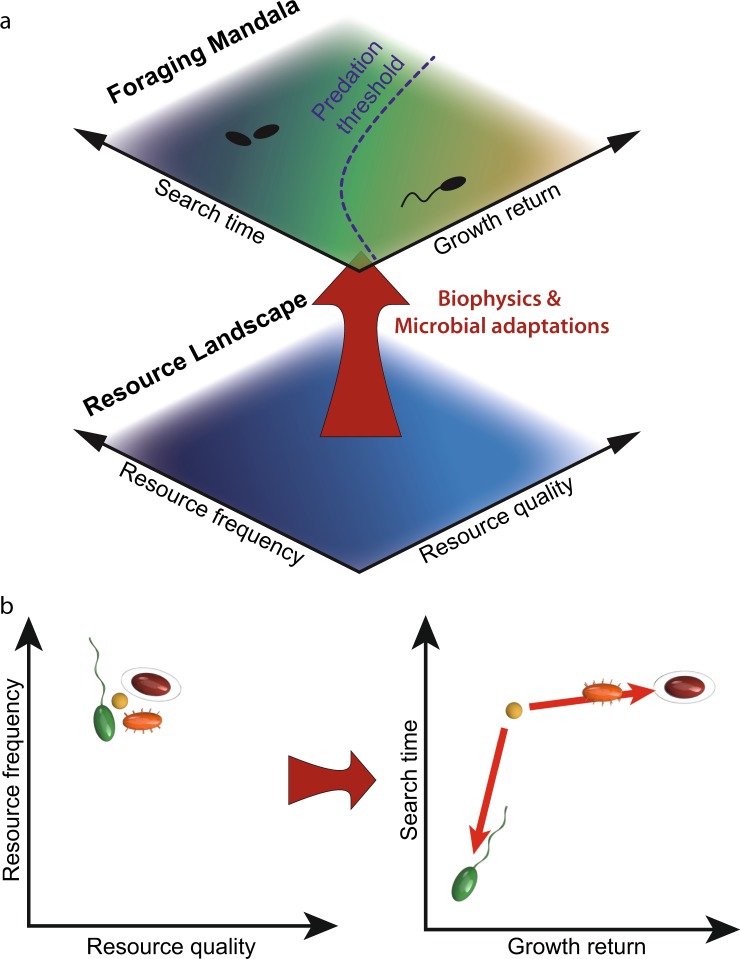
Schematic illustration of the foraging mandala for aquatic microorganisms. **a** The resource landscape (bottom plane), characterized in its simplest form by the frequency and quality of resource patches, is not the only determinant of foraging. Rather, biophysical processes and biological adaptations ‘map’ the resource landscape onto a foraging landscape – or foraging mandala (top plane). The latter consists of two axes – search time and growth return – that encompass foraging performance. The color-coding indicates how favorable a given condition is for microbial growth, with lighter colors corresponding to more favorable conditions (light blue in the resource landscape, yellow in the foraging mandala). **b** Organisms that inhabit the same resource landscape (same location in the resource landscape plane, at left) but have different foraging adaptations (planktonic – yellow; swimming – green; attaching – orange; attaching and biofilm forming – red) will occupy different regions in the foraging mandala (at right), representative of their different foraging performances in that resource landscape. For example, swimming reduces the search time and thus shifts the position on the mandala downward, while attachment and biofilm formation enhance the growth return and thus shift the position on the mandala rightward

The outcome of microbial foraging, in the water column as in other environments, hinges on the resource landscape: the quantity, composition, and spatiotemporal distribution of resources. It is a challenge to quantify the quality of the aquatic resource landscape for microorganisms and further to parameterize it in order to interpret the different nutrient acquisition strategies exhibited by microorganisms. Intuitively, a resource landscape can be described in terms of two general metrics, related to the spatiotemporal ‘frequency’ of occurrence (how many patches there are per volume) and the quality of resources in them. For example, in a water column containing particulate organic matter, one could quantify the concentration of particles (the ‘frequency’ of the patches) and the average amount of available carbon in each particle (the quality of the patches). These two resource landscape metrics are not new: because of their generality, they are cornerstones of macroecology and feature, for example, in the intermediate disturbance hypothesis [7], the patch dynamics concept [8] and in optimal foraging theory [9]. However, these two loosely defined metrics, which are purely related to features of the resource landscape, cannot encompass the foraging performance of diverse microorganisms, which are further determined by the organisms’ adaptive behaviors (Fig. 1a). For example, it would be a challenge to pin down a practical metric for resource ‘quality’ that encompasses all the different sources of carbon and other nutrients that microorganisms can utilize. For this reason, the foraging mandala views the features of the resource landscape through the lens of the organisms’ adaptive behaviors, resulting in foraging metrics that allow the consistent and comparative interpretation of a variety of environments and foraging adaptations.

Two fundamental parameters determine a microorganism’s foraging success: the average search time the microorganism spends in seeking the next resource patch, and the average growth return (yield) it obtains from a resource patch. The growth return is quantified in terms of the number of new foraging cells that the microorganism produces from exploiting a patch. Importantly, the growth return differs from the growth rate of a foraging organism, as it takes a holistic view of the growth resulting from an interaction with localized resources, independent of the duration of the interaction and of the search time (unlike the average growth rate). Search time and growth return are heavily influenced – but not solely dictated – by the features of the resource landscape (i.e., the frequency and the quality of patches). However, converting the features of the resource landscape into search times and growth returns requires understanding the biophysical processes constraining foraging at the microbial scale.

The microbial foraging mandala that we propose (Fig. 1a) utilizes growth return and search time as its two axes to provide the flexibility needed to account for a broad range of foraging adaptations of aquatic microorganisms, while corresponding in general, intuitive terms to the frequency and resource content of patches in the resource landscape. The first axis, the growth return, is an organism-centric formulation of the resource quality that allows for diverse microbial traits and interactions to be incorporated. For example, marine snow particles are composed of a complex palette of compounds, and different bacteria have adaptations for utilizing different compounds as carbon sources: comparing the benefits and costs of metabolizing multiple carbon sources or specializing on individual ones becomes conceptually straightforward with the growth return metric. The second axis, the search time, for some microorganisms (e.g., motile ones) captures the period of active behavioral search for the next patch, whereas for others it describes the ‘waiting’ until the next encounter with a resource. As we will show, the biophysical lens of these two axes – growth return and search time – makes it possible to consider in a unified framework processes as diverse as the bacterial utilization of transient nutrient hotspots, bacteria-algae symbioses, host-phage interactions, bacterial nutrient acquisition in homogeneous waters, the colonization of marine snow particles, and predation by zooplankton.

The axes of the foraging mandala therefore combine the effects of both the environment and microbial foraging adaptations. Returning to the example of marine particles, for a given concentration of particles and a given resource content per particle, the foraging outcome – specifically, the time between two particle encounters by the same microorganism and the growth return the microorganism acquires from a particle – will be very different for different microorganisms and will depend strongly on their foraging adaptations (Fig. 1b). Motile bacteria will have greatly decreased search times compared to non-motile bacteria, hence motility shifts a microorganism’s position downward on the mandala, but will incur metabolic and proteomic costs that can reduce growth return. Attachment and biofilm formation will increase the duration of interactions with particles and can thus enhance growth return, hence shift a microorganism’s position rightward on the mandala. In this way, organisms that inhabit the same resource landscape nonetheless experience different conditions for growth.

**Fig. 2:**
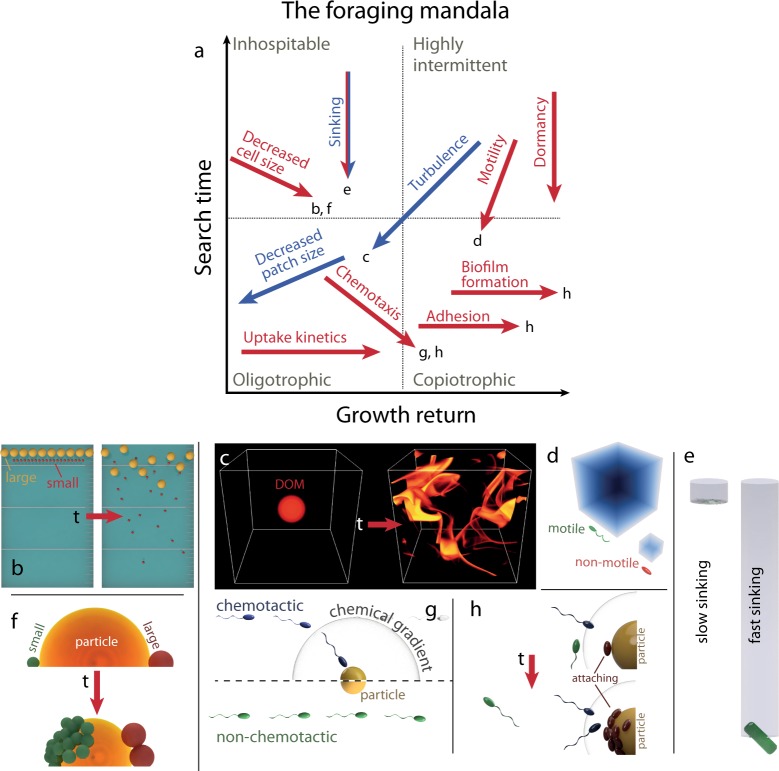
The effect of different biophysical processes and biological adaptations on the location of a given foraging strategy in the foraging mandala. **a** Each factor affects one or both axes of the mandala. The quadrants in panel **a** identify different growth regions, corresponding to inhospitable growth conditions (top left), oligotrophic growth (bottom left), copiotrophic growth (bottom right), and highly intermittent conditions (top right). The arrows corresponding to each element denote the direction in which that element moves the position of a forager in the mandala. The red and blue arrows indicate biological factors and environmental elements, respectively. The “sinking” arrow is represented in both colors because both resources and microorganisms can sink. The spatial location of each arrow is only indicative of (and not limited to) the growth region where that element may be mostly expected. Elements are further illustrated individually in panels **b** through **h**. **b** Size directly affects search time for non-motile microorganisms because smaller objects (red) diffuse further than larger objects (yellow) in a given time (see also Eq. 1). **c** Turbulence affects both search time and growth return by deforming resource patches into a plethora of filaments, for which the search time as well as the growth return are lower than for the original patch (reproduced from ref. 20). **d** Motile microorganisms (green) explore greater volumes of water per unit time compared to non-motile microorganisms (red), resulting in a considerable reduction in the search time. **e** Both sinking and rising reduce the search time because the associated flow enhances encounter rates (see also Eq. 2). **f** Smaller cells (green) attain greater growth returns for the same resource compared to larger cells (red). **g** Chemotactic microorganisms (blue) need only encounter the cloud of solutes around a resource (e.g., a particle) in order to rapidly move to the resource, thus reducing the search time compared to non-chemotactic microorganisms (green). **h** Retaining position relative to a resource patch, whether by chemotaxis (blue cells) or attachment (purple cells), enhances growth return, as purely randomly motile microorganisms (green) rapidly lose position relative to the patch

To more intuitively understand the foraging mandala, before considering further examples, it is useful to first describe the different regions of the mandala (Fig. 2a). In the lower left region (small search times, small growth returns) resources are found quickly but provide little return for each patch or interaction. A region near the origin corresponds to oligotrophic growth, in which small ‘patches’ of individual molecules with correspondingly small growth returns, representing a chemically homogenous environment, are nonetheless encountered sufficiently frequently to support growth. The lower right region of the mandala (small search times, large growth returns) corresponds to resource patches that are rich and encountered frequently, thus favouring copiotrophic behaviors. The upper left region of the mandala (large search times, small growth returns) is the most inhospitable, corresponding to long periods of time between encounters with meager resources. In the upper right region (large search times, large growth returns), individual resource patches are rich but encountered very infrequently: this is possibly the least understood region of the mandala, and one expects behaviors that favor the thorough utilization of individual patches or the occurrence of dormancy to bridge the long search times. Generally, the growth rate and resilience of microbial populations increase towards the lower right region in the mandala.

In the following, we exemplify the use of the mandala by describing the mechanisms underpinning several microbial foraging adaptations and presenting simple calculations to evaluate their impact, then illustrate how this understanding can be usefully framed in the context of search time and growth return.

## Biophysical insights into foraging adaptations of aquatic microorganisms

### Search time

When considering search times in foraging (Fig. 2a, *y*-axis of the mandala), a large body of literature on encounter rates can be leveraged [10]. The reference microbial behavior that provides a baseline against which microbial traits are best evaluated is that of a non-motile microorganism lacking any active search behavior. In this situation the ‘search’ time, i.e., the time that intervenes between when the organism experiences consecutive resource patches, still depends on features of the organism (e.g., size) and of the environment (e.g., concentration of patches, presence of flow). The search time can be quantified by borrowing formulations for encounter rates between two objects in a fluid medium. In the simplest case in which one object is the microorganism and the other is a small resource patch (e.g., a small particle), the encounter rate *Q* (number of resource patches encountered by the forager per unit time) is determined by the relative diffusive motion of the two objects and is quantified as

1 $$Q = 4\pi \left( {r_{\rm{F}} + r_{\rm{R}}} \right)\left( {D_{\rm{F}} + D_{\rm{R}}} \right)C_{\rm{R}}$$

where *r*_F_ and *r*_R_ are the radii of the forager and the resource, respectively (both assumed spherical), and *D*_F_ and *D*_R_ are their respective diffusivities. From Eq. 1, the search time can then be computed as *T*_S_ = 1/*Q*. The resource concentration *C*_R_ represents the number of concurrently available resource patches in a unit volume (imagine taking a snapshot of that volume and counting the resource patches). This accounts for situations in which resources are transient, either because they correspond to short-term release events (e.g., lysis of phytoplankton cells) or because they move through the fluid (e.g., sinking particles). In cases where the encounter does not always succeed (e.g., a bacterium that does not always attach to the particles it encounters), the encounter rate must be multiplied by an efficiency parameter *α* (between 0 and 1), measuring the probability of successful attachment [11]. Any forager or resource surrounded by water moves diffusively due to Brownian motion, which can be quantified with the Stokes-Einstein equation, $D = \frac{{k_BT}}{{6\pi \eta r}}$, where *k*_*B*_ is Boltzmann’s constant, *T* is the temperature in degrees Kelvin, and *η* is the dynamic viscosity of water. For large objects, this is typically negligible. Even for an *r*_F_ = 0.5 µm radius bacterium, the diffusivity by Brownian motion is *D*_F_ = 4×10^−13^ m^2^/s. Frequently, the resource is considerably larger than the forager, so the diffusivity of the resource and the size of the forager can be neglected in Eq. 1, resulting in the simplified expression $T_{\rm{S}} = 1/Q = 1/\left( {4\pi {r}_{\rm{R}}D_{\rm{F}}C_{\rm{R}}} \right)$ for the search time.

As the simplified expression shows, decreasing cell size moves an organism’s position on the mandala downward. For example, a non-motile, *r*_F_ = 0.5 µm radius bacterium ‘searching’ for the surface of *r*_R_ = 100 µm radius particles that occur at a concentration of 10^4^ per liter, has a search time of ~6 years. Passively searching by Brownian diffusion (or, more appropriately, ‘waiting’) for 100 µm particles is thus clearly a poor foraging strategy. This calculation can be extended to account for realistic distributions of particle sizes [12]. Microorganisms could in principle reduce this search time by being smaller (Fig. 2b), yet not enough to result in useful search times: even viruses (*r*_F_ = 25 nm) only encounter 100 µm particles every 115 days. It is important to point out that these long search times do not equate to non-motile bacteria necessarily being rare on particles. If we change our perspective to that of a 100 µm radius particle ‘searching’ for non-motile, 0.5 µm radius bacteria present at a concentration of 10^4^ cells/ml, then the particle ‘waits’ on average 2 days for one encounter with a non-motile bacterium.

Foraging in a dilute resource environment with a low but essentially constant resource concentration *C*_R_, typical of oligotrophic bacteria in the ocean (Fig. 2b, lower left ‘oligotrophic’ region of the mandala), can be interpreted as ‘searching’ for individual molecules. This strategy can be quantified through the same formulation (Eq. 1), though now the search time is determined by the size of the forager *r*_F_ and the molecular diffusivity of the resource molecules *D*_R_, resulting in $T_{\rm{S}} = 1/Q = 1/\left( {4\pi r_{\rm{F}}D_{\rm{R}}C_{\rm{R}}} \right)$. Small molecules have a diffusivity on the order of *D*_R_ = 10^−^^9^ m^2^/s so that a foraging bacterium (*r*_F_ = 0.5 µm) encounters one molecule every 0.02 ms for *C*_R_ *=* 10 nM, typical for example of nitrate concentrations in strongly oligotrophic waters [13]. Such brief search times (with correspondingly smaller payoffs) suggest very different foraging adaptations, but are again captured in the foraging mandala.

Swimming, or other forms of microbial motility such as buoyancy, can under the right circumstances greatly shorten the average search time for resources, thus shifting an organism towards the lower regions of the mandala. Intuitively, motility allows organisms to explore water volumes faster, which reduces search times. However, this intuition becomes more complicated for microorganisms due to the particular physics at the microscale. For microorganisms that have limited directional persistence, motility results in a diffusive pattern [14, 10], where the long-term behavior mirrors that of Brownian motion and is less efficient in exploring new volume than steady linear motion. With such diffusive swimming, the encounter rate with resource patches can again be described with Eq. 1, only with a considerably higher diffusivity (on the order of *D*_F_ = 10^−^^9^ m^2^/s, i.e., >1000-fold larger than Brownian diffusion) that originates not from Brownian motion but from swimming [14]. This vast difference in the diffusivity of non-motile and motile bacteria is at the origin of their vastly different search times, because (for large resources) we saw that the search time varies with the inverse of the forager’s diffusivity. For example, under the same conditions considered previously, motility shortens the average search time of a 0.5 µm radius bacterium for a 100 µm particle from 6 years to 22 h, making encounters with particles a massively more frequent occurrence and a more viable foraging strategy. In a similar manner, motility can enhance the encounter rate with predators (see section 3 for a discussion of predation), potentially offsetting some of the benefits from the reduction in search times.

When the target resource of a search is moving in a consistent direction with respect to the searching organism, such as for particles sinking (or rising) through the water column (Fig. 2e), the flow of fluid that this movement causes can decrease the search time, shifting the forager to the lower part of the mandala (Fig. 2a). The magnitude of this decrease can be computed using standard results from fluid mechanics. Consider a sinking particle of radius *r*_R_, large enough that its diffusivity can be neglected, sinking at speed *U*. The nature of the flow created by the particle is determined by the Reynolds number, Re = *r*_R_*U*/*v*, where *v* is the kinematic viscosity of water. For most marine snow particles, size and speed are small and the Reynolds number is significantly smaller than 1, implying that viscous forces dominate over inertial forces and preventing certain types of (‘inertial’) encounters [10]. The effect of the flow generated by the particle on the search time is controlled by a second dimensionless number, the Peclet number Pe = *r*_R_*U*/*D*_F_, which measures the relative importance of transport driven by fluid flow compared to transport by diffusion. For low Reynolds numbers (Re < 0.1), the encounter rate of an organism searching diffusively for (large) sinking particles is enhanced to [15]

2 $$Q = 4\pi r_{\rm{R}}D_{\rm{F}}C_{\rm{R}}\left[ {1 + \left( {1 + 2\rm{Pe}} \right)^{1/3}} \right]/2$$

and thus the search time 1/*Q* is reduced by the presence of flow (note that when the Peclet number is very low, the search time is unaffected by flow). For an *r*_R_ = 100 µm radius particle sinking at *U* = 33 m/day [16], one has Re = 0.04 and Pe = 9.8×10^4^, and the search time for particles by non-motile bacteria (*r*_F_ = 0.5 µm) is reduced by a factor of almost 30 relative to the case of non-sinking particles. In the case of swimming bacteria, the same sinking rate of the particle only causes a 3-fold additional reduction in the search time beyond the 3000-fold reduction caused by swimming itself – motility is thus considerably more important than sinking in setting the search time. In contrast, for a different scenario, where bacteria (e.g., swimming at *U* = 45 µm/s) are the ‘resource’ and viruses are the foragers (*r*_F_ = 25 nm), one has Re = 2×10^−5^ and Pe = 2.8, so that the fact that the bacterium swims reduces the search time of a virus for the bacterium only by a factor of 1.4.

Swimming, buoyancy, and reduced cell size all decrease the search time by allowing cells to explore larger volumes of water per unit time. Separately, the search time is also influenced by the size and frequency of patches, with larger targets providing higher chances of encounter and thus a reduced search time. Chemotaxis – the ability to direct motility along chemical gradients – is a bacterial foraging trait that affects search times by increasing the effective size of resource patches (Fig. 2g, h). For example, instead of having to encounter a particle itself, a chemotactic organism will more rapidly encounter the (larger) cloud of chemicals that surrounds the particle and then migrate towards it. The reduction in search time depends on the size of the cloud that the organism can sense, which in turn depends on the organism’s chemotactic sensitivity [17] and on the local flow [18]. The effect can be considerable, as the cloud of chemicals that the microorganism can sense can be of the order of 100 times the size of the particulate resource itself [19]. Even with a far more modest assumption of a four-fold increase in the effective resource size due to the chemical cloud, chemotaxis is predicted to reduce the search time of a motile bacterium for a 100 µm particle (following the earlier example) from 22 h to 5.5 h. Unlike undirected motility, which affects only the search time, chemotaxis can also affect the growth return of motile microorganisms (Fig. 2h, see next section). Chemotaxis thus moves the position of a forager at an angle on the mandala (Fig. 2a).

Turbulence, like swimming, sinking, or rising, also moves foragers lower in the mandala towards reduced search times and more frequent encounters with particulate resources (Fig. 2a, c). Turbulence is the motion of the fluid characterized by eddies (vortices) across a range of scales. At large scales the randomness of turbulence can lead to diffusive-like motion [16]. However, microorganisms are typically smaller than the smallest eddy size (the ‘Kolmogorov scale’, ~0.1–3 mm, depending on the turbulence intensity; [20]), so they experience turbulence only as ‘simple shear’, a linear variation of fluid velocity over distance. A Peclet number can again be defined (though somewhat differently; [15]), and governs the reduction in the search time of a forager seeking a particle: for Pe » 1, T_S_ is a factor of 1.49Pe^−1/3^ lower in the presence of turbulence compared to quiescent conditions. For non-motile, *r*_F_ = 0.5 µm radius bacteria searching for *r*_R_ = 100 µm radius particles, one has Pe = 25 (turbulence intensity of 10^−6^ m^2^/s^3^) and the foragers’ search time is thus reduced 2-fold due to turbulence. Additional effects such as the rotation of the particle by turbulence can also be taken into account [21, 15].

For resources that are localized patches of high solute concentration, such as those left behind during grazing events, turbulence alters the nature of the resource landscape (Fig. 2c). For patches larger than the Kolmogorov scale, turbulence stirs a patch into many filaments [20] (Fig. 2c), thus changing the resource landscape from a single, rich patch into one characterized by many smaller, weaker patches. Turbulence can thus alter both the search time and the growth return for microorganisms foraging on dissolved resources, shifting foragers’ position on the mandala down and leftward (Fig. 2a).

The duration of a resource patch is an important feature of a resource landscape that can alter (also greatly) the impact on the search time of adaptations such as swimming. Hotspots generated by lysing diatoms or grazing copepods have lifetimes governed by diffusion of solutes back to background concentrations. Even marine snow particles, oil droplets or other substrates that take long to degrade may saturate with bacteria so that new bacteria encountering the substrate cannot attach. The finite duration of a resource needs to be accounted for when calculating the concentration of concurrently available resource patches that an organism can encounter (*C*_R_ in Eq. 1). In addition, the transience of resources highlights another encounter mechanism, which requires an alternative computation of the search time: organisms encounter the resource patch if they are within its region of influence when the patch is created. This apparently trivial mechanism – being at the right place at the right time – can in some cases be the dominant one in determining the search time. This is the case for chemotactic bacteria foraging for the nutrient pulses created by lysing diatoms, where it was recently shown [19] that the number of bacteria initially inside the area of influence of the lysis event (a 2 mm radius sphere) was more than 10 times larger than the number of bacteria that encountered this area of influence over the lifetime of the event (~10 min). The number of cells arriving versus those present at the start become comparable when the duration of an event is longer than (*r*_R_)^2^/(3*D*_F_), independent of the mean concentration of bacteria (where *r*_R_ is the footprint of the resource patch and *D*_F_ the diffusivity of the forager). In resource landscapes that are highly transient, the search time of a microorganism can thus be determined by the probability of a resource patch arising within an appropriate neighborhood of the microorganism, and is independent of adaptations such as motility.

### Growth return

For the proposed foraging mandala, we consider the average number of new cells that each cell produces from its interaction with one resource patch – what we call the growth return (*x*-axis of the mandala, Fig. 2a). The growth return can be as small as −1, if the original forager always dies in the resource interaction before generating any offspring. Three categories of adaptations can affect a microorganism’s position relative to the growth return axis of the mandala: the capability to engage the resource, the efficiency in uptaking the resource and the efficiency in turning the resource into biomass (specifically, new cells). Other elements such as competition or predation can reduce the growth return by reducing access to the resource or causing mortality: these detrimental effects that occur during an interaction with a resource would similarly be included as factors that diminish the growth return. Designing experiments to directly quantify the number of new cells produced on each patch is technically challenging, thus few experimental results are available that quantify the influence of a trait on growth return. However, as we describe below using selected quantitative examples, one can often deduce from indirect measurements and simulation results in which direction on the mandala a given trait moves the growth return.

The capability to thoroughly exploit a patch becomes especially important as the forager occupies the upper part of the mandala (Fig. 2a), where the search time for patches is long. Maintaining position in a microscale environment is a surprisingly challenging task, because diffusion – in addition to mediating the encounter with new patches – also works to disperse cells away from a localized resource. A fast swimming 0.5 µm marine bacterium has ~2500-fold higher diffusivity than its non-swimming counterpart. If both cells find themselves in the center of a 100 µm radius resource patch, the swimming cell will find itself outside the patch (by random motility) after on average <2 s, compared to 70 min for the non-motile cell (Fig. 2h). Once a resource patch has been found, remaining at the resource will have a significant impact on the growth return.

Aquatic microorganisms have evolved a number of adaptations to maintain or improve their position relative to a resource (Fig. 2h). Chemotaxis, in addition to potentially reducing search times, allows swimming bacteria to remain in local maxima of chemoattractants [22] such as those found near a lysing diatom [19] or within nutrient filaments created by turbulence [20]. Notably, in turbulence-stretched nutrient filaments, patches are transient and no solid surface is available for attachment. As a result, if the resource concentration threshold defining a patch is similar to the threshold at which bacteria can chemotactically respond to gradients, there can be very little benefit to the search time from chemotaxis. Instead, chemotaxis allows a cell to migrate from the fringe of a nutrient filament to the regions of highest concentration, or to remain in those regions for longer. This increases the exposure of a chemotactic cell to the nutrients in the filament (both in time and concentration) and increases the average growth return from an interaction. In contrast, a non-chemotactic motile cell that encounters the edge of a nutrient filament is as likely to leave the filament as it is to move closer to its center, and as a result the typical interaction with a filament will result in less exposure to the nutrients than for a chemotactic cell. Direct numerical simulation results suggest that chemotactic cells which exploit nutrient filaments created by turbulence can experience varying degrees of growth advantage, from 60 to 140% more than non-chemotactic bacteria, depending on the speed of chemotactic migration [20]. In a different context, non-attaching chemotactic bacteria have been found to obtain more than double the growth return from a ten-minute resource pulse originating from a diatom lysis, compared to non-motile cells [19]. Whether chemotaxis results in a positive or negative growth return depends on whether the added growth during an interaction with a patch outweighs the cost of the motility and chemotaxis machinery. While little is known about the precise magnitude of these energetic costs, we highlight that the cost of chemotaxis [23] is almost negligible compared to that of swimming (estimated at ~0.5–3 new cells worth of carbon per day [20]).

A second adaptation for retaining position at a particulate resource is adhesion. Microorganisms often stably adhere to surfaces, thus preventing diffusive separation, through a number of mechanisms, including hydrophobic interactions and cell-surface appendages such as pili, flagella, and stalks [24]. The attachment can vary in duration, from transient to permanent [25]. By adhering to a marine particle, bacteria ensure for themselves durable exploitation of the particle’s organic matter. For a marine *Vibrio* lineage in the presence of model marine particles, adhesion and biofilm-forming phenotypes have been found to afford an ~2-fold larger growth rate compared to non-attaching cells and to result in longer interaction durations [26]. In this scenario, the growth return can be estimated as the growth rate divided by the frequency of interaction with a patch. Adhesion is also critical for the prolonged interactions in marine microbial symbioses, such as those of UCYN-A cyanobacteria and picoplankton algae [27], and in the many cases in which marine bacteria are observed to be conjoint [28]. The increase in growth returns associated with these adaptations moves microorganisms rightward in the mandala (Fig. 2a), though the growth return of a stable symbiosis remains difficult to quantify. Securing a stable association with a particulate resource, however, also carries its costs, in terms of genomic and energetic investment (e.g., attachment proteins) and of the risks of being transported by particles to unfavorable environments. These costs, which counter the rightward shift on the mandala, also remain poorly quantified.

Adhesion further allows microorganisms to access resources that are unavailable to planktonic cells. This is, for example, the case when direct contact is required for the enzymatic degradation of a particulate resource, as occurs for membrane-bound enzymes. With secreted enzymes [29], attachment is also beneficial, as ‘degradation at a distance’ (as would be performed by purely planktonic cells) would have poor yield due to the loss of enzymes by diffusion and advection.

Some bacteria enhance their attachment, and growth return, by forming biofilms, cementing their foothold on surfaces through the production of extracellular polymers [30, 31]. Biofilms further increase the growth return from attachment by altering their local physical environment. Considering, for example, marine particles, as particulate matter is enzymatically degraded and becomes dissolved, a large fraction can escape from the particle [32]: forming a biofilm enhances the growth return by reducing the loss of the resources (‘solutes’) after their dissolution. At the same time, biofilms have also been found to promote the activity of the enzymes by buffering pH, favoring quorum sensing, and reducing predation [33]. Chemotaxis, adhesion, and biofilm formation thus all move foragers rightward in the mandala, towards increased growth return from the interaction with a resource patch.

Most microorganisms obtain carbon and other nutrients that are needed for growth through diffusion from their surroundings. This immediately implies that uptake (when diffusion-limited) is enhanced by high local concentrations and at the same time susceptible to environmental (flow) and biological factors (e.g., competitors consuming the same resource) that may reduce the resource’s local concentration. Substrate molecules that contact the surface of a microorganism must specifically encounter transporter proteins in order to be brought into a cell and metabolized, which under certain conditions can also be limiting. The uptake of substrates as a function of their ambient concentration is frequently summarized by Michaelis–Menten enzyme kinetics, with different rate constants capturing the different inter-membrane transport conditions specific to each substrate [34]. There is a trade-off in transport between maximum uptake rate and strong affinity to substrate [35, 36]: the former favors growth under highly intermittent high-quality patches whereas the latter favors uptake in low, steady nutrient concentrations. For the purposes of increasing growth return – and so again moving them rightward in the mandala – organisms may tune uptake kinetics at the level of individual transporters (e.g., altering the substrate affinity) or of the entire organism (e.g., altering the number of transporters). Microorganisms also express different transporters, governing which (and how many) substrates they have access to. In the right resource landscape, these adaptations can greatly improve the growth return, but if the landscape changes they become a dead weight.

Cell size is one of the physical properties relevant to the growth return. Smaller cells have higher surface-area-to-volume ratios. Since uptake on rich resources can be limited by the number of transporters and thus by the cell’s surface area [37], whereas the biomass required to produce a new cell is proportional to the cell’s volume, hypothetical microorganisms that differ solely in having a larger size will have a longer doubling time. This longer doubling time allows more time for resources to escape or be exploited by competitors, lowering growth return. The increased biomass needed to generate one offspring also directly impacts growth return for larger cells, since growth return is quantified in units of new cells. Even for a scenario with a modest size difference – two spherical bacteria, one having twice the radius of the other – the growth return of the smaller bacterium will be eight times that of the larger one for the same amount of resource taken up (Fig. 2f). For example, phytoplankton, which are constrained externally in daily light exposure and might have weaker correlation between size and carbon fixation, show evidence for a negative relation between growth return and cell size [38]. Importantly, size rarely varies in isolation for microorganisms: large heterotopic bacteria often exhibit greater maximum growth rates compared to smaller ones [39], due to differences in uptake kinetics. As a result, despite the above biophysical arguments for the impact of size on growth return, it is difficult to perform rigorous empirical tests to quantify the effect of size alone. However, when nutrients are limiting, this effect of size can generate significant evolutionary pressure for an organism to streamline its genome and reduce its size [40]. For these reasons, shrinking cell size without factoring other adaptations moves foragers rightward on the mandala, improving the growth return. At the same time, every adaptation in microorganisms that involves additional genes incurs a growth return cost in terms of increased size, potentially larger than just the increased genome if it also results in new intracellular proteins.

Gain in growth return can be offset by losses due to predation and competition while interacting with a resource. Interactions between microorganisms are apparent from the reproducible succession observed on model particles [41]. This results in a range of possible adaptations to increase the growth return. One example is that of secondary degraders and of cheaters in microbial communities on marine particles: these bacteria take up dissolved resources made available via hydrolysis by primary degraders and avoid the cost of producing degradation proteins, thus increasing their growth return on the particle [42]. A second example are defensive adaptations to remove competitors or reduce competition for a resource. Defensive mechanisms reduce the foragers’ growth return by reducing the fraction of the resource that it can use directly for new biomass, but in exchange may afford a privatization of the resource that causes enhanced growth return. While well-known ecological concepts in this domain can provide guidance for the types of impact that social or community interactions have on growth return, obtaining quantitative assessments of such adaptations through experiments and modeling is more difficult and remains a frontier in microbial ecology.

Many of the microbial adaptations for improving growth return are independent of the search time, and have been presented as net gains in the growth return. This corresponds to purely horizontal rightward arrows in the mandala (Fig. 2a). One could thus ask why most microorganisms do not possess the entire arsenal of these growth-return-enhancing adaptations. Here it is important to recognize that a microorganism does not consistently interact with only one type or size of resource. Returning to the case of adhesion, the growth return cost of the ability to adhere is present even when the microorganism exploits an ephemeral nutrient burst due to a lysing diatom to which it does not adhere, but the benefit is only maximized when the resource is particulate. The relative availability of resources suitable for a certain adaptation will then dictate whether that adaptation represents a net gain or loss in terms of growth return.

## Towards fundamental principles of microbial foraging in aquatic environments

Our purpose in this Winogradsky review was to propose to the community of aquatic microbial scientists a biophysical framework that illustrates how different foraging adaptations allow aquatic microorganisms to utilize the often heterogeneous resource landscape of the water column in oceans, lakes and other water bodies. The resulting foraging mandala shows how a variety of physical and biological factors can be ultimately distilled into two fundamental metrics of foraging performance – the search time and the growth return. These two independent metrics depend on the biological adaptations of the organisms, the environment, and the physics constraining the interplay of the two. Although we have often selected our examples from processes involving bacteria in laying the foundation for the foraging mandala, this framework applies more broadly to marine microorganisms. Microbial prey, symbiotic partners, and even individual molecules can each be viewed as localized resources in the foraging mandala.

The mandala is sufficiently general to account for elements of foraging that may at first not appear to be related to search time or growth return, such as the mortality risk associated with foraging. Whether attached to a particle, clustered by chemotaxis, or accidentally in the vicinity of a resource patch, microorganisms can experience a different mortality risk in patches than in the search phase between patches. Dense populations and heightened microbial activity in a patch can increase the risk of viral infection [43] and predation. Biofilms on particles are themselves rich resources for larger foragers [44]. This predation risk effectively corresponds to a decrease in the average growth return of a bacterium from a patch and can thus also be accounted for in the proposed framework. For example, if a fraction *P*_1_ of the bacteria that engage with marine snow particles are lost to viral infection before dividing and a fraction *P*_2_ of bacteria on the particles are scraped off by copepods before dividing, then the expected growth return *G* of bacteria on the particles will be reduced to *G*′ = (1−*P*_1_)(1−*P*_2_)*G*. In this manner, both the benefits and the costs of defensive mechanisms can be further incorporated into the growth return, determining the position of a given strategy along the *x*-axis of the mandala.

**Fig. 3:**
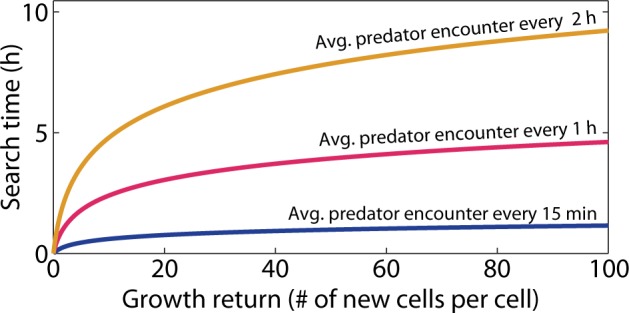
Predation thresholds in the foraging mandala. Three curves define regions (below the curves) of the foraging mandala in which the growth return is sufficient to enable at least one cell on average to successfully reach the next resource. Above the curve, predation on average wipes out a population before the next resource is found. The threshold depends on the predation rate (labeled), which in turn is a function of the predator concentration

Microorganisms experience mortality not only when utilizing a resource, but also during the search phase of foraging, for example through encounters with predators. The impact of mortality during searching can be visualized in the mandala by defining a lower bound for the growth return, defined as the minimum growth return sufficient to offset the mortality during the search phase. A strategy is not sustainable if the search time is so large that on average less than one cell from those dispersing from the previous patch (i.e., the original cell plus those generated in terms of growth return) survives predation during the search. Given a fixed predation rate, the cells dispersing from the previous resource patch and searching for a new patch will decline exponentially in number over time, at a rate governed by the predator encounter rate [10]. This threshold defines a population survival region in the mandala (Fig. 1a), which varies with the frequency at which predators are encountered (Fig. 3). The saturating shape of these predation thresholds indicates that even for very rich resources with high growth returns, foragers cannot afford too long a search time if there are predators.

As with any simplified representation, there are many elements of the ecology of aquatic microorganisms that are not captured by the foraging mandala. Generality is part of the foraging mandala’s strength in bringing together a variety of microbial adaptations and environmental features, but it is important to recognize what the mandala does not account for. One such element is growth rate. In order to capture in the mandala a wide variety of resource interactions, we defined the growth return without any notion of time. In other words, the growth return only measures the total amount of resource that a microorganism converts into new cells from a patch, and not the rate (amount of resource divided by time) at which it does so. As a result, the average growth rate of a microbial population having a certain foraging adaptation can only be determined from the growth return with additional information on the typical duration of the interaction with a resource patch (e.g., the time that a bacterium stays attached to a particle). For example, if two bacterial species both have a search time of 6 h, but one an average growth return of eight cells and the other of two cells, then one cannot immediately conclude that the first species has a higher overall growth rate, since growth rate depends on the duration of the interaction with a resource patch. Importantly, however, this still allows for the direct graphical comparison of population dynamics among microorganisms that are competing for the same resources and that have similar interaction durations.

A second element not captured by the mandala is cell-to-cell heterogeneity. The mandala is based on foraging outcomes that are averages of a potentially broad range of outcomes that individuals can experience. However, in every search and growth phase there will be some individuals that significantly outperform the average, through either biological variability or pure environmental stochasticity. For example, assuming the encounters of a population of microorganisms with their resource patches are random and independent, with an average search time of 1 h, then 20% of the searches will take <14 min and 1% will take <1 min. Similar heterogeneity applies to growth return when the latter is dependent for example on a range of available resources (e.g., particle sizes). A small, random, ‘lucky’ subset of individuals in a population of foragers may have a disproportionate effect on population dynamics and microbial interactions. This sort of cell-to-cell heterogeneity is necessarily obscured by any population-level generalization, yet might be important in driving population dynamics and social phenomena such as bet hedging and division of labor.

The foraging mandala presents a high-level abstraction for a universal element of microbial life: nutrient acquisition. Its primary purpose is to provide a unified view of the diverse microbial adaptations, for comparison and quantification, as well as to highlight how the resource landscape is modulated by those adaptations in order to determine foraging performance. The foraging mandala also naturally defines a notion of a ‘foraging distance’ between different microbial populations in the same environment, which may lead to practical predictions in microbial ecology. For example, a community of microbes that interact on the same marine snow particle may occupy different locations in the foraging mandala despite their physical proximity, for instance because both non-motile and motile cells are part of the community. The foraging mandala framework would predict that the composition of such a particle-degrading community is more stable to environmental fluctuations (e.g., frequency and size of particles, temperature, ambient flow) if community members are in close proximity on the foraging mandala, as the foraging of all of them would be impacted similarly. From another perspective, two microorganisms that utilize the same resource and are closely positioned in the foraging mandala (i.e., have a small “foraging distance”) are likely to be in direct competition. For mature communities in stable environments, one would therefore expect that populations utilizing the same resource are characterized by some minimum foraging distance between each other.

The foraging mandala may also help identify functionally similar groups of microorganisms (those having similar search times and growth returns), refine environmental categories, and predict ecological consequences from shifting environments (such as phytoplankton blooms). Categories of microorganisms such as oligotrophs or copiotrophs have conventionally been defined based on their ability to grow in different concentrations of dilute resources or based on their genomic characteristics [45]. The foraging mandala presents an opportunity for practical distinctions in oligotrophic/copiotrophic microbial life strategies that are based on their utilization of typical resource landscapes.

While there is a large body of knowledge, both theoretical and experimental, that can be leveraged to quantify search times, it is comparatively harder to evaluate the impact of different adaptations on growth return based on current knowledge. This points to the importance of measurements of the net influences of each adaptation (e.g., chemotaxis, adhesion, biofilm formation, enzyme production, toxin production) and of a better understanding of the cost of these adaptations in environments not suited to them. Fortunately, differential growth return due to a specific adaptation is experimentally measurable. For example, the growth of biofilm-forming vs. planktonic populations of very closely related *Vibrio* populations has been compared using artificial marine particles as sole carbon source [26]. Though often difficult, such differential quantifications are important and should be performed more broadly in order to better understand the quantitative influence of different microbial adaptations to the resource landscape of aquatic environments. The foraging mandala presents a framework for such measurements and a blueprint for quantitative understanding of microorganism–resource interactions at the level of single cells, from which the vast global flow of carbon and other elements starts.
